# Effect of dog-related parameters on the flexion test outcome: A large cohort retrospective study on physiological and orthopedic pathological-related factors

**DOI:** 10.3389/fvets.2022.1064795

**Published:** 2022-12-15

**Authors:** Diane Grosjean, Evelien De Bakker, Amélie Mugnier, Franck Forterre, Jimmy Saunders, Bernadette Van Ryssen, Yves Camiel Alice Samoy

**Affiliations:** ^1^Faculty of Veterinary Medicine, Ghent University, Merelbeke, Belgium; ^2^Département NeoCare, Ecole Nationale Vétérinaire de Toulouse (ENVT), Toulouse, France; ^3^Vetsuisse Faculty, University of Bern, Bern, Switzerland

**Keywords:** dog, orthopedic examination, joint assessment, flexion test, pathological factors

## Abstract

**Objectives:**

This retrospective study evaluates the dog-related factors of variation influencing the outcome of the flexion test (FT), when performed to localize pain to a joint area, on a large group of canine orthopedic patients.

**Materials and methods:**

The selection criteria for this retrospective study were dogs undergoing a FT in a referral orthopedic clinic between 2009 and 2020 with a complete medical record. The canine FT, described in a previously published protocol, was performed on dogs presented with an orthopedic problem. In summary, a dog's joint, identified as suspected of an orthopedic problem according to the clinical examination, was flexed for 1 min before walking 15 m on a hard and even surface. The FT was considered positive if the lameness increased after the application of the FT and negative when it remained unchanged. Statistical analysis was performed to determine which of the following criteria could influence the outcome of the flexion test: age, gender, neutered status, weight category, tested joint and initial lameness score.

**Results:**

Over 1,161 patients' files were collected and analyzed for this research. The FT showed 82.8% (95%IC: 80.5–84.9) of true positives and 17.2% of false negatives. None of the patient's intrinsic characteristics influenced the outcome of the test (age, gender, neutered status, and weight category). The orthopedic parameters, such as the initial lameness score and the tested joint, showed to have a statistically significant influence on the outcome of the test.

**Clinical relevance:**

The FT is an easy-to-perform technique presenting reliable results on most joints. This test presents an interest when performed in addition to a complete orthopedic examination to localize pain to a joint area. Only the orthopedic pathological-related parameters such as the lameness score and the tested joint seem to influence the outcome of the FT. The FT is not influenced by the physiological-related characteristic of the patient (age, weight category, sex, and neutered status).

## Introduction

The flexion test was first introduced in equine veterinary practice as a specific physical examination developed for orthopedic health and locomotion evaluation. This flexion test (FT) is routinely performed during gait assessment, lameness evaluation and pre-purchase examinations (1–3). Previous research in horses' orthopedics agrees on this tool's sensitivity to assess pain in a joint area, but not on its specificity to diagnose a joint pathology (4, 5). In this species, the FT is defined by a pain response triggered after the flexion of a joint to its physiological maximum range of motion for a defined period (3, 6). This position creates a compression of the joint structures and a stretching of its surrounding soft tissues which can trigger a pain reaction (4, 7). To evaluate the result of the test, the horse is immediately walked on a hard surface on a straight line to evaluate his gait, and graded according to its duration expressed in meters (8, 9).

The flexion test (FT) is also a popular tool used in human medicine where it is utilized to assess pain sensation in the elbow, wrist and cervical region (10–12). This is a major difference between human and veterinary FTs. For example, to assess neck pain related to an alteration of the neuromotor control of the deep flexors muscles of the neck, a cranio-cervical FT was specifically developed in human medicine (10). This test aims to identify deep cervical muscle dysfunction as a likely cause of cervicogenic headaches in humans when in combination with upper cervical symptomatic joint dysfunction (10). The modified passive neck FT used in human patients with chronic cervical non-specific pain was found to have excellent inter- and intra-examiner reliability (94 and 81% agreement, respectively) (13). Other tests were developed to diagnose specific pathologies. For example, two tests were described to screen for cubital tunnel syndrome: the shoulder internal rotation test and the elbow flexion test. The shoulder internal rotation test when applied pre-operatively to patients with cubital tunnel syndrome found a specificity of 100%, and a sensitivity of 80%, which was statistically significant when performed for 10 s (*p* = 0.001) (11). On the opposite, the elbow FT revealed a low sensitivity of 36%, but 93% of the patients presented a pain reaction (11, 12, 14). Other flexion tests are also described for pain evaluation specific to a pathology. To test for lateral epicondylitis, the Mill's test (extension of the wrist) was developed (12). To test for pathologies affecting the wrist region, the Phalen's wrist FT is designed for patients with carpal tunnel syndrome (15) and the Finkelstein's and Eichhoff's tests were defined to diagnose the DeQuervain's tenosynovitis (12, 16).

The FT is a clinical tool commonly used as a complementary method to assess lameness in horses. Unlike human flexion tests, where pain sensation is assessed by the reaction from the patient, veterinary flexion tests aim to assess pain through the lameness enhancement induced by the FT. This test is complementary to the palpatory and gait examination and can be performed easily in routine orthopedic examination (1, 2). The mechanism proposed behind the FT response is centered on the pain reaction to a flexed position held from 5 s to 3 min (17), and to the continuous tension and compression of a joint, including the surrounding soft tissues (9). A shorter duration of 5 s has also been described in equine medicine, but this was less likely to produce positive tests (17). When performing a FT, the blood pressure in the subchondral bone vessels may increase (7, 9) and the mechanoreceptors of the joint area can detect a modification in their stretch (18). These receptors include the Ruffini endings and free nerve endings as major receptors in the shoulder and knee joint structures, including the joint capsule, muscle tendons, intra-articular and collateral ligaments (19, 20); the Golgi-organ tendon and muscle spindles at the musculotendinous junction (21) and the nociceptive specialized Schwann cells in the skin (22). The consecutive mechanical deformation on the cell membrane allows entrance of Na^+^ ions into the cell creating a depolarization and generation of a nerve receptor potential (21). This can create a pain response observed as a temporary lameness scored with the use of a visual analog scale (VAS) (6).

Equine veterinarians first defined this test by its duration and the force applied to the joint (6, 8, 9). Previous research in horses agrees on this tool's sensitivity to assess pain in a joint area, but not on its specificity to assess joint pathology (4). Equine research classified factors influencing the outcome of a flexion test as examiner related-factors, physiological horse-related factors, and pathological horse-related factors. Among physiological horse-related factors, the effect of age, gender, weight, height and fetlock range of motion was studied (6). The age and gender showed in this species a significant influence on the outcome of the FT: older horses and mares being more prone to produce positive tests (*p* < 0.05 for both of these parameters) (6, 9). On the opposite, joint range of motion, weight and height had no influence on the FT results in the equine species (6). The examiner-related factors, defined as: positioning, time and force applied, have shown to be of great importance on the outcome of the test (7, 8). Therefore these studies concluded a standardization of the test is needed to insure repeatability, resulting in the creation of a flex-test tool (9). The flex-test tool was developed for the measurement of the traction forces occurring during the FT of distal metacarpophalangeal and interphalangeal joints. This tool contains pressure sensors and is applied by the user on the dorsal hoof wall when performing a FT. This tool was developed for research purposes to assure constant and appropriate pressure during the FT but was abandoned for practical use in a clinical environment (9).

The FT has also been used in dogs (23). When the basic orthopedic examination for joint effusion, thickening, range of motion, crepitus and pain is inconclusive, the FT can be incorporated into the clinical evaluation (23). This method is easy to use and accessible to all small animal clinicians with no additional equipment costs. The FT applied on dogs has been described in the literature as a flexion of a single joint to a full range of motion. This position is held for 1 min before the dog is released to walk immediately on a straight line on a homogeneous and hard surface of 15 m. This first definition available in canine medicine doesn't consider the force applied to the joint unlike some protocols described in equine medicine (9, 23). This is because of practical limitations in dogs. A preliminary study described the use of this tool in addition to the orthopedic examination as promising (23), but data are still lacking regarding the clinical accuracy of this tool when tested on a larger group of dogs. Inspired by equine colleagues, the FT was translated to dogs according to the protocol previously described in the literature (23). In this study, the flexion test offered a significant lameness score increase (*p* < 0.001) and a specificity of 100% after it was performed on a sample population of orthopedic lameness cases (23). The FT has also been shown as providing a large score of 81.5% of true positives among a small group of patients presenting an orthopedic pathology (23).

Inspired by the study from our equine colleagues mentioned above (6), the aim of this present study is to explore if any dog-related factors influence the flexion test result. These will be evaluated when the FT is applied to a large group of canine patients. The goal of this retrospective study is to select physiological dog-related factors and pathological dog-related factors that could be identified of potential influence on the flexion test result such as: age, weight category, sex, neutered status, joint tested and initial lameness score.

## Materials and methods

The canine flexion test has routinely been used for 20 years in the refferal orthopedic department of Ghent University as part of the orthopedic examination.

This offers the opportunity for this study to present a retrospective design: clinical records from all orthopedic patients presented to the academic department mentioned above for lameness complaints (not lame, to severe upon presentation) between 2009 and 2020 were collected.

Selection criteria for this retrospective study were dogs undergoing a FT of any joint from the appendicular skeleton, with the presence of a clinical diagnosis based on gait evaluation and orthopedic examination, medical imaging records (radiographic images and/or ultrasonographic evaluation and/or computed tomographic images), and arthroscopic results when available. The definitive diagnostic was reached with the support of the complementary examination methods described above. This means that only a population of orthopedically diseased dogs was selected for further statistical analysis.

Each patient was assigned to one of the diplomates in veterinary sports medicine (ECVSMR) defined as the observer. After a complete clinical and palpatory examination, one joint of the appendicular skeleton was determined as the most suspected of an orthopedic abnormality. The parameters taken into account upon palpatory examination were: limb muscle atrophy, joint effusion, decreased range of motion or pain at joint manipulation (indifferently at flexion and/or extension) for each patient by the observer (24). The use of the flexion test is defined as a complementary clinical tool to allocate pain to a joint area (4, 23). In this context, this test is used as a second line of examination, after the palpatory evaluation, and before further complementary examination (such as medical imaging). Therefore, when a doubt existed about the exact pain location within a limb, a flexion test was performed by the observer on one or more joints starting with the least painful joints. All joints were evaluated separately with a washout period in between two tests. The washout period was defined as the time necessary for the lameness to return to the baseline level (lameness score at the first presentation). The result of the flexion test obtained using this method supports the area of choice for further medical imaging investigation. Consequently, straight forward cases, with no doubt about the location of the orthopedic issue after palpatory examination didn't undergo a flexion test, and therefore were organically not retained among these selected retrospective cases that underwent a FT in this clinical context. The limbs that underwent one or multiple FT as defined above were included in this study, but data regarding which cases received multiple FT as well as their respective results and joints tested were also not retained among these selected retrospective cases and subsequently evaluated with those patients that only received one FT. Indeed, as defined in the selection criteria of this study, the medical imaging results provide a definitive diagnosis by describing a lesion as being the most relevant finding within this limb. Therefore, the FT result could be compared to the diagnostic findings for the considered joint.

The FT method applied was previously described in the literature (23): first, the initial gait at presentation was evaluated by the same observer on a hard and even surface of 15 m, recorded, and scored on the four points visual analog scale (VAS) described by Brunnberg (not lame, mild, moderate or severe lameness; Table 1) (25); second, on the dog in a standing position, only the affected joint of interest was flexed to maximum range of motion in an isolated manner and held in this position for 1 min (timed with a chronometer) by the observer according to the previously published guidelines (23); third, the dog was walked again on the same hard and even surface of 15 m taking the first steps into account. Gait velocity and gait pattern should approximate that of the initial examination: a fast walk, or at trot, to optimize the visualization of the lameness. This lameness was scored on the VAS by the same observer. This score was compared to the original lameness score to conclude as a positive or negative test. A positive FT result demonstrate an increase of one point or more after the FT when a negative test is defined by an unchanged lameness score after the FT (23). A remark should be made about the dogs initially presented with a 4/4 lameness score upon presentation. Those patients could be considered positive on the flexion test if they evolved from intermittently non-weight bearing to constantly non-weight bearing according to the set of definitions presented in Table 1.

**Table 1:** Four points visual analog scale described by Brunnberg.

**Lameness score**	**Definition**
Not lame	The patient is not affected
Mild lameness	The patient is barely affected, and consistently weight bearing
Moderate lameness	The patient is affected, and inconsistently weight bearing
Severe lameness	The patient is severely affected and intermittently to constantly non-weight bearing

Finally, the dogs underwent medical imaging examination according to the methods defined above. This helped defining the definitive orthopedic diagnosis for each patient. A true positive result was defined as a positive FT obtained on a patient presenting a confirmed lesion on the tested joint area by medical imaging method. On the opposite, those who presented a negative FT on a joint area with confirmed lesions were considered as false negatives.

To resume the set of definitions described above, the defined statistical analysis inclusion criteria for this study are: diseased patients presenting a lameness suspected from orthopedic origin upon palpatory examination on one most painful joint within an identified limb. The patient must later be confirmed with an orthopedic problem located on a joint area using an imaging technique. These criteria must be matching the area of the flexion test performed during the clinical examination. Consequently, only one performed flexion test per case was retained for this retrospective study.

According to the above set of definitions and to the goals defined in the introduction, statistical analysis will be applied to the tested group of patients to evaluate if any of the following parameters could have an influence on the outcome of the test: age, sex, neutered status, weight category, baseline lameness score, and tested joint.

### Statistical analysis

A binary logistic regression was fitted to explore the factors influencing the result of the FT when applied on joints with an orthopedic pathology. The outcome was a binary variable indicating when the FT was positive or negative. The candidate explanatory variables included general characteristics of the dog: sex (male/female), neuter status (neutered/entire), age (continuous, in years) and weight category. Depending on adult weights, canine breeds were classified into four sizes: small (<10 kg), medium (10–25 kg), large (26–45 kg) and giant (>45 kg) (26). In addition, two orthopedic parameters were considered: the affected (and tested) joint (carpus/elbow/hip/shoulder/stifle/tarsus/digits) and the lameness score (not lame/mild/moderate/severe). The overall fit of the model was evaluated by the dispersion parameter, which should be close to 1.

Statistical analysis was performed using R software [(27); version 4.1.2]. Results with *p*-values < 0.05 were considered as significant. Statistical uncertainty was assessed by calculating 95% binomial confidence intervals (95%CI).

The dispersion parameter was 0.9 implying there is no over-dispersion of the population.

## Results

Between March the 25th 2009 and December the 12th 2020, 13,520 patients were presented to the referral orthopedic department of X with lameness complaints. Of these patients, 1,576 had received a FT (11.65% of all orthopedic patients). Of those, only 1,161 met the specific selection criteria of this study of a flexion test performed in combination with a medical imaging orthopedic diagnostic confirming pathology in the joint which received the FT. The patients excluded didn't have a complete medical record (*n* = 61), or they were lacking medical imaging complementary examination (*n* = 185), or didn't have a definitive diagnosis (*n* = 96), or didn't have a clear flexion test result reported in the clinical file (*n* = 73).

**Figure 1:**
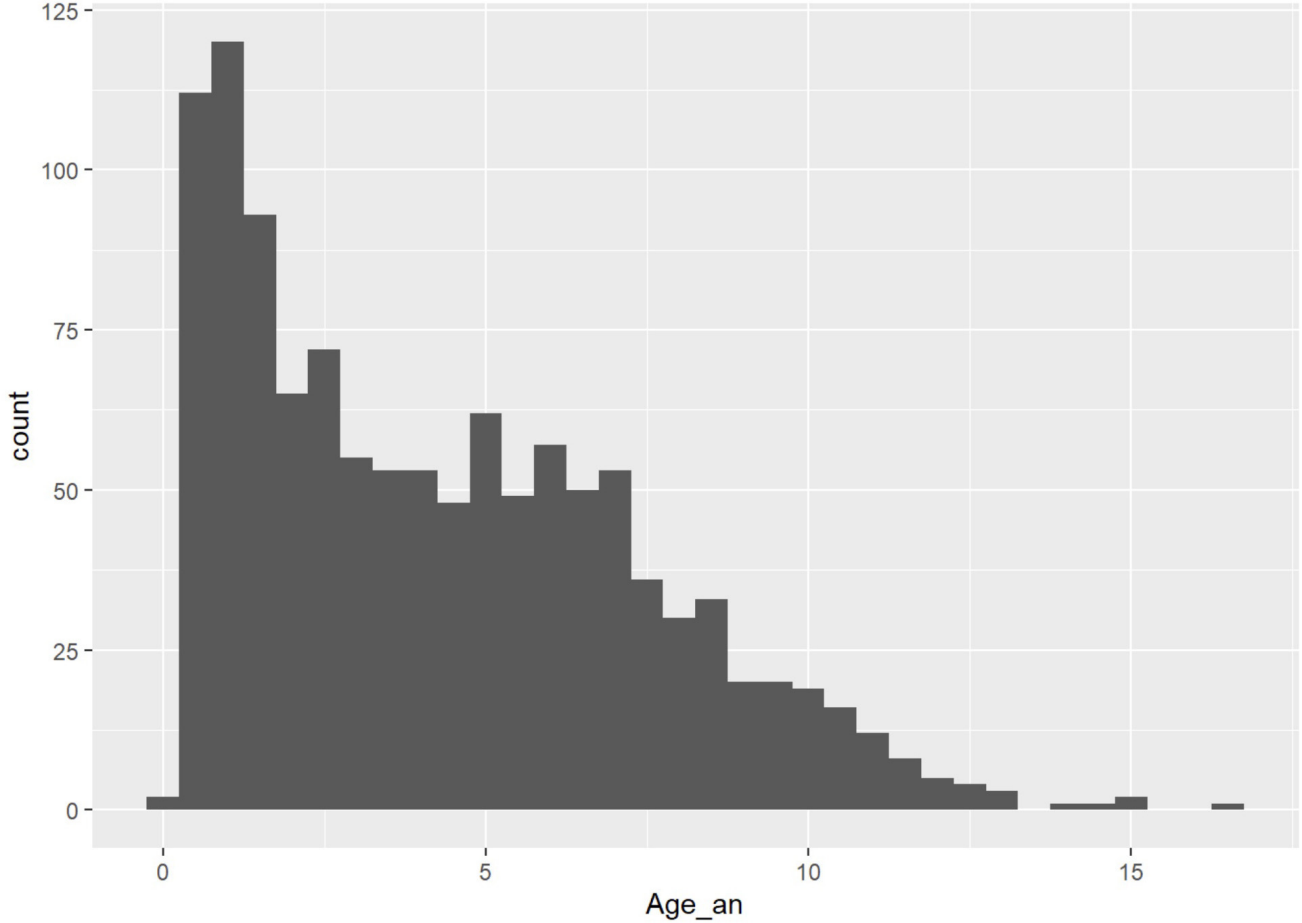
Age dispersion among the population (*x*, age in years; *y*, number of patients).

According to the selection criteria, the studied population gathered 612 males (52.7%) and 549 females (47.3%). Among those, 395 patients were neutered (34%), and 766 were intact (66%). The age of the patients ranged from 0.1 year (1.2 months) to 16.7 years (median 3.8 years; Figure 1). The dogs were classified into weight categories for statistical analysis purposes: 106 small dogs (under 10 kg; 9.1%), 272 medium dogs (10–25 kg; 23.4%), 619 large dogs (25–45 kg; 53.3%) and 164 giant dogs (over 45 kg; 14.1%). One-hundred and forty breeds were represented among those categories.

From the 1,161 patient's files fitting the selection criteria, 867 dogs had a front limb FT (74.7%) and 294 underwent a hind limb FT (25.3%). The tested joints were the shoulder (101 tests, 8.7%), the elbow (693 tests, 59.7%), the carpus (32 tests, 2.8%), the hip (69 tests, 5.9%), the stifle (165 tests, 14.2%), the tarsus (32 tests, 2.8%) and the digits (69 tests, 5.9%). Among those 40 tests were performed on the digits of the front limb (57.9%) and 29 tests were performed on the digits of the hind limb (42.1%).

The patients were spread into four categories of lameness scores: not lame (238 patients, 20.5%), mild lameness (458 patients, 39.5%), moderate lameness (375 patients, 32.3%), severe lameness (89 patients, 7.7%).

The FT showed 82.8% (95%IC: 80.5–84.9) of true positive results (961 cases) against 17.2% of false negatives (200 cases). Given the study design that exclusively selected for cases with confirmed pathology in the joint that received the FT, no false positives (positive flexion test in the absence of a confirmed associated pathology) nor true negatives (negative flexion test on a normal joint) were identified.

The results of the logistic regression showed that both considered orthopedic parameters (lameness score and joint evaluated) were influencing the response to the FT with a statistically significant difference (*p* < 0.001 for both parameters).

**Figure 2:**
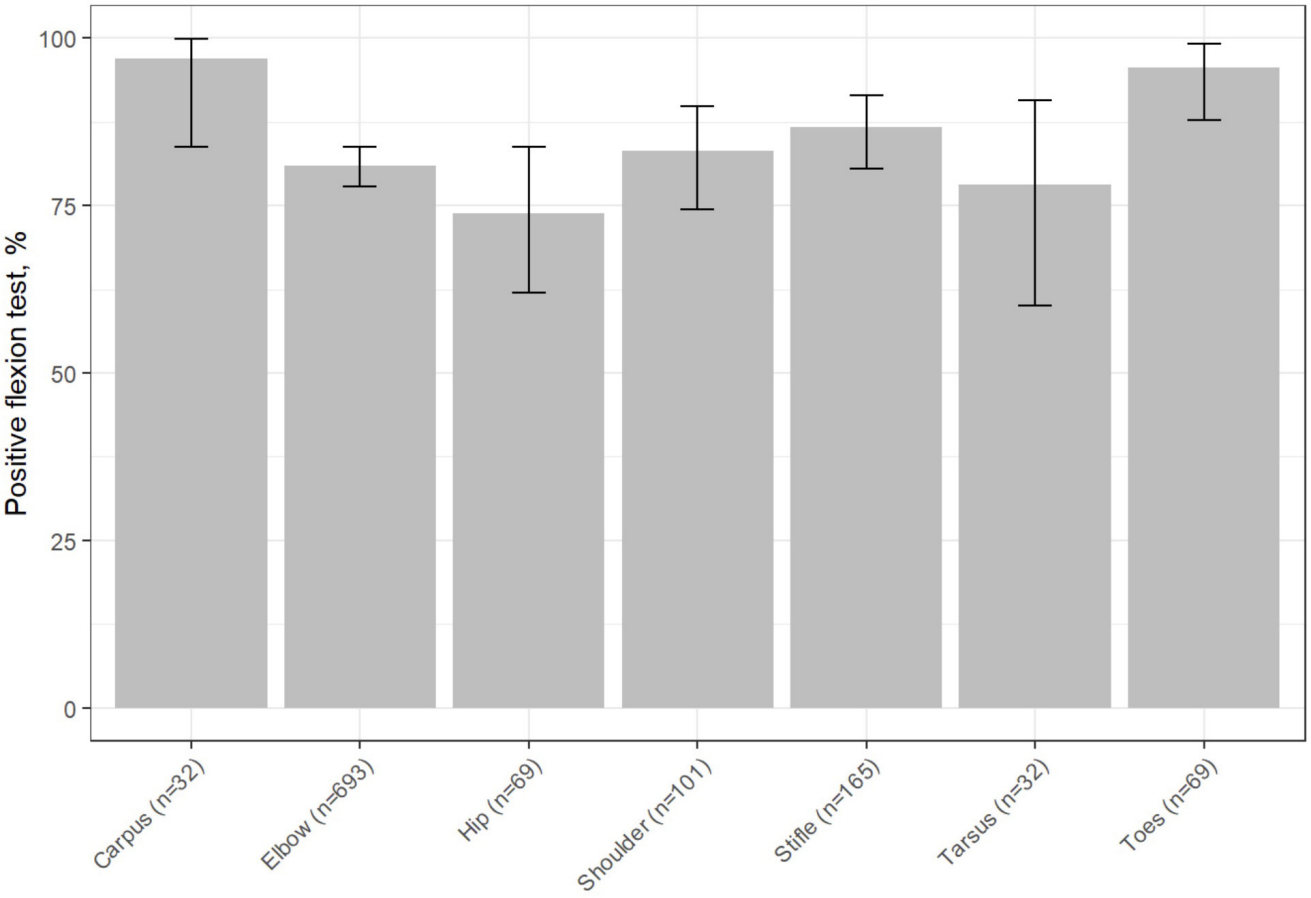
Percentage of positive FT results considered by joint area [*x*, joint area (and an indication of the number of positive results); *y*, percentage of positive FTs]. The T bar indicates the 95%IC.

**Figure 3:**
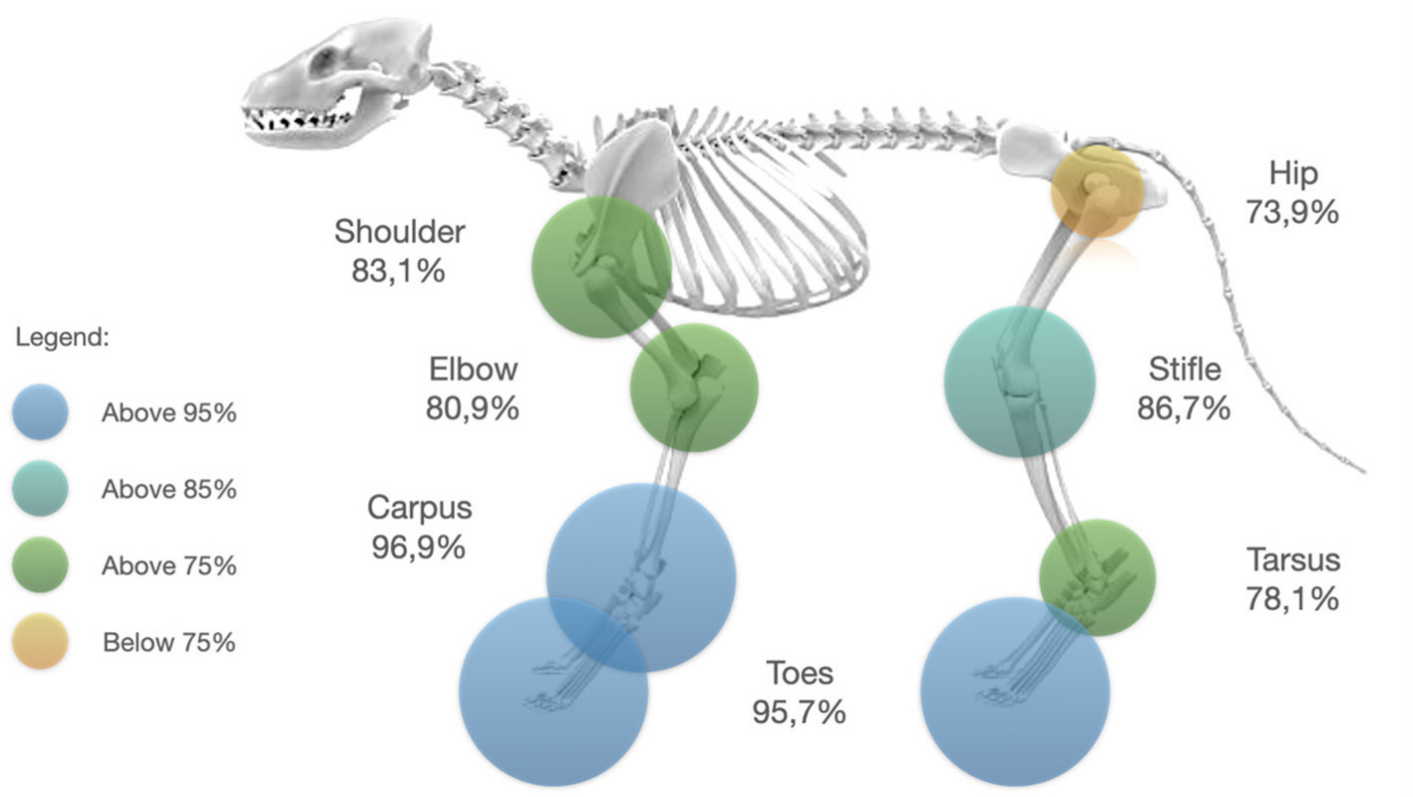
Percentage of true positive flexion test result exposed by joint area. The circles represent the proportion of true positives recorded for the considered area. The colors represent gradually the true positive percentage result reached for each joint area.

With 96.9% of true positives (95% CI: 83.8–99.9; Figure 2), the carpus was the joint with the highest amount of true positive results followed by the digits with 95.7% (95CI: 87.8–99.1; Figure 2). On the opposite, the least accurate results were obtained when the hip joint was evaluated (73.9% of positive results, 95% CI: 61.9–83.7; Figure 2). The other joint areas showed similar true positive results rates: 80.9% for the elbow (95CI: 77.8–83.8; Figure 2), 83.1% for the shoulder (95CI: 74.4–89.9; Figure 2), 86.7% for the stifle (95CI: 80.5–91.4; Figure 2), 78.1% for the tarsus (95CI: 60.1–90.7; Figure 2). These results are illustrated on the Figure 3.

**Figure 4:**
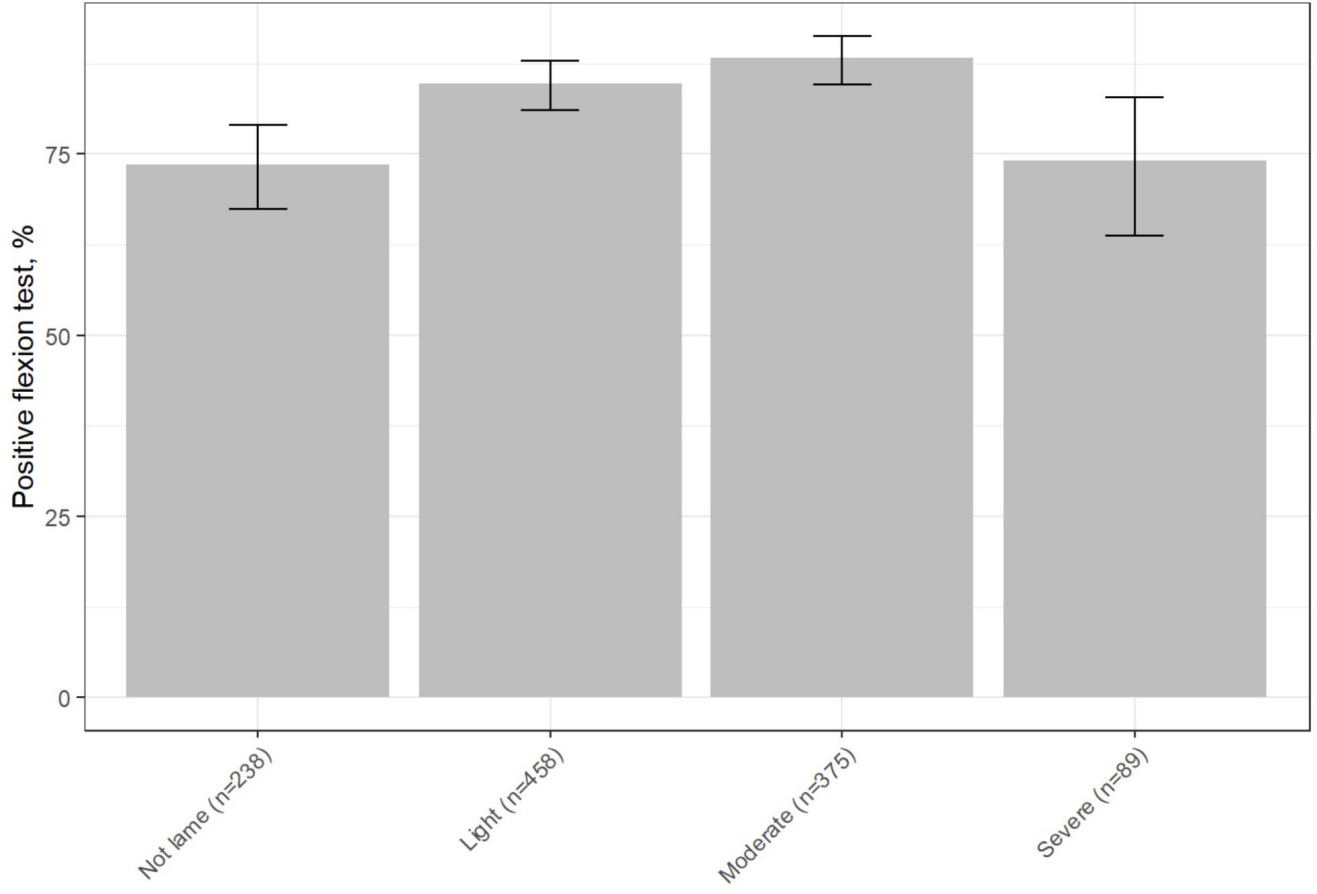
Percentage of positive FT results depending on the initial lameness score result. [*x*, initial lameness score (and an indication of the number of positive results); *y*, percentage of positive FTs]. The T bar indicates the 95%IC.

Regarding the lameness score, most of the false negative results to the FT were obtained when dogs were initially not lame or when they presented a severe lameness score. Indeed, 26.5% and 25.8% of false negative results were respectively reported for the not lame and severe lameness scores patients. On the other hand, only 15.3% and 11.7% of false negatives were reported for the mild and moderate lameness scores, respectively (Figure 4). Figure 4 shows the percentage of positive FTs obtained when considering the initial lameness score of the patients: 73.5% for the non-lame dogs (95CI: 67.4–79.1; Figure 4), 84.7% for the mild scores (95CI: 81.2–87.9; Figure 3), 88.3% for the moderate scores (95CI: 84.6–91.3; Figure 4), 74.2% for the severe score (95CI: 63.8–82.9; Figure 4).

None of the dog's physiological-related factors (age, sex, neutered status, and weight category) appeared to influence the result to the FT on a pathological joint (*p* > 0.1). The data regarding physiological-related parameter and statistical analysis are reported in the Table 2.

**Table 2:** *P*-value obtained for each one of the dog's physiological-related parameters after statistical analysis.

**Parameter**	***P*-value**
Weight category	0.89
Age	0.44
Sex	0.76
Neutered status	0.63

None of the results is statistically significant.

## Discussion

The FT was first introduced as a promising tool in addition to the orthopedic palpatory evaluation to localize the origin of a lameness on canine patients (23). Information regarding the use of this test on a large study sample is lacking. The present study contributes to increase the knowledge about this test when it is used in a clinical environment. Indeed, this test offered a result of 82.8% of true positives when applied to a large cohort of orthopedic patients. This result reinforces the previous conclusion available in the literature. Indeed, the FT was initially described to produce true positive results in 81.5% of cases when tested on a small sample population (23). On this same study, false negative FTs were estimated as 18.5%. Again, this result is confirmed on this large cohort study with a refined result of 17.2%. Furthermore, this observation reinforces the indication to use the FT as an implementation to the orthopedic examination in combination with other routinely used tools such as gait evaluation, palpation, joint distension, range of motion and pain (24). When the orthopedic examination is suggestive of an orthopedic issue on a joint area, and the FT is positive: complementary examination such as medical imaging should be performed to confirm the nature of the problem in this area.

The characteristics of the patients examined in this study showed a statistically significant influence of the dog's orthopedic pathological-related factors such as the lameness score and joint area on the outcome of the FT on dogs. In equine literature, the studied effect of the orthopedic pathological-related factors was limited to the fetlock (metacarpophalangeal joint) range of motion and this showed no influence on the outcome of the FT (6). This highlights a difference between the equine and canine species regarding the influence of orthopedic pathological-related factors on the flexion test outcome.

Furthermore, the results exposed by this canine study seems to outline a tendency for the FT to be overall more consistent on the joints of the fore limb compared to the joints of the hindlimb. One of the hypothesis, could be that the hindlimb is prone to consensual movements between the stifle and tarsus, and this could maybe be of some influence on the FT results.

Alternatively, the physiological-related factors of the patient such as age, sex, weight category, and height were studied in the equine species, and some demonstrated an influence on the outcome of the FT. The age and gender of the horses showed a significant influence on the outcome of the FT. Indeed, older horses and mares produced more positive tests than younger horses and geldings (6). However, these results were not confirmed on dogs. The outcome of the FT was not influenced by the age, sex, neutered status, or weight category of the dog. Furthermore, the dimension parameters available on both species can be compared. Authors suggest that the weight category of a dog may be considered as an equivalent parameter to the weight and height of a horse. In that way, the results provided in the equine literature regarding the influence of the dimensions of the patient are also confirmed in dogs to have no influence on the outcome of the FT.

The initial lameness score showed a significant influence on the FT outcome on dogs. Indeed, the dogs with mild or moderate lameness were more likely to produce positive tests. Whereas dogs initially not lame or affected with a severe lameness were less likely to become positive after applying the FT. This result reinforces previously published data obtaining the very same observation (23). In this previous study, the discussion highlighted the limitations of the four points visual analog scale (VAS) suggesting the use a more precise way of scoring instead. This will be further discussed as one of the limitations of this study.

The joint area was also proven as having a significant influence on the FT outcome on dogs. The carpal joint and digits were producing the most accurate positive results with a detection of more than 95% of the cases. This result presents a limitation since the fore limb digits and hind limb digits were considered as only one group of joint in the statistical analysis. The other joints (elbow, shoulder, stifle, and tarsus) provided a reliability close to the overall reliability of the FT detecting an average of 80% of the cases presenting an orthopedic pathology. This result implies that a positive FT of those joints is highly suggestive of a pathology. Therefore, the FT result is an indication for further medical imaging investigation. Opposingly, the hip joint presented the lowest reliability only detecting pain in 73.9% of the cases. Therefore, cautious interpretation should be made when a negative FT occurs on a hip region, especially when the previous palpatory orthopedic examination is suggestive of a pathology. A lead of improvement to test the hip joint would be to introduce a hip extension test instead of a hip flexion test to explore if this adapted method provides more reliable results.

The joint area is defined as a pathology-related factor directly influencing the outcome of the FT. Therefore, the joint area, when painful, can be considered as the source of the clinical lameness. Protocols including intra-articular anesthesia to prove the accuracy of the primary source of the lameness could be considered to refine these findings (28). Using this method, the definitive source of the animal's lameness could confirm the suspicions exposed by the FT. This would help to avoid any confusion between a potential medical imaging incidental finding (for example joint osteoarthritis on a carpus) and a pathology being the real source of pain (for example an elbow infection). This points out the need for further research, such as breaking down each type of joint pathology to know if this parameter is influencing the FT response. Furthermore, a lead of refinement for the FT could be, as exposed in human medicine (10–12, 14, 16), to know if the use of a specific positioning could be targeting certain types of soft tissue injuries, always more nebulous to diagnose upon palpation in veterinary medicine.

Room for development of the FT is available by selectively testing joints on an affected limb. Indeed the canine FT could be used to discriminate a painful joint from others within a limb considering the description of the technique (23). Therefore, a practical suggestion would be to start by testing the least suspected joint to end with the most suspected joint on the limb. Then, the identification of the most painful joint should be possible with refinement. Indeed, the observer can compare the lameness scores and pain reactions, such as lameness or behavioral changes, collected after performing the FT on different joints from a limb. This application for the technique could be of practical use in a clinical environment to localize the area responsible of pain, and most likely the origin of a lameness. This application for the FT requires further research to examine if such a testing method could result in more false positives due to the repeated testing and manipulation.

This advice should be adapted on the hind limb taking into account its biomechanical singularity (23). Indeed, the extensor digitorum longus is described to have an influence on the consensual movement of knee and tarsus (29). Then a recommendation for the FT of the stifle is to perform it first, and then perform a FT of the tarsus when the clinical examination is indicative of a problem on one of those localizations (23).

Further investigation is needed regarding the possible use of a FT on several joints of the same limb as a discriminant to localize the painful area in doubtful cases. Considering the design of this study, this situation occurred in an unknown number of cases. Therefore, several joint testing is a limitation to the interpretation of the results of this study.

The results of this study on a large cohort of canine patients also presents an interest in terms of evaluation of animal welfare when performing a FT. The FT aims to trigger pain to a joint area suspected of a pathology. The FT should be avoided in cases where the painful joint is abundantly clear and appropriate diagnostic imaging can be selected without a doubt. Unfortunately, in clinical conditions, the orthopedic examination is not always providing such clear information, such as in elbow cases, largely represented in this retrospective study. Therefore, the flexion test is of clinical interest to help confirm the painful area to support the area of choice for further medical imaging investigation and avoid several exposures to sedations and/or radiations when a doubt about the location of the problem subsists. The pain generated by the FT is only temporary. This unpleasant feeling for the patient is fading away after a few minutes (23). In addition, in this retrospective study, only a minority of cases were excluded because of behavioral issues due to pain during the manipulation. This helps to confirm the good tolerance of the method described in previous research (23). However, given the uncontrolled, retrospective design, selection bias likely also occurred.

As exposed in the indications for the FT, this test should be only performed in case of the inability of the observer to precisely locate the problem after the palpatory examination. For ethical considerations, it seems obvious that a test meant to trigger pain to locate a potential orthopedic problem should only be performed if no other examination at this stage could help to point out the area of the problem. In case of such a situation: this is when the FT is meant to help the practitioner. This is an additional step before selecting the areas to be examined on further medical imaging investigation if this step was not already possible after the initial palpatory examination.

There are several limitations to this retrospective study. First, the patients selected were all dogs presented in a referral orthopedic center for lameness diagnosis. Referral patients are mostly patients with challenging orthopedic conditions to diagnose in a first line practice. This can create a bias regarding the proportions of joints diagnosed in this study with the use of a FT. Indeed, a large majority of elbow cases are reported over all the other joints. Furthermore, cases that offered obvious changes upon palpatory examination didn't receive a flexion test and were not included in this study. The excluded cases may have biased results and underrepresent the true sensitivity of the FT. An additional bias to be mentioned is that the cases underwent medical imaging examination according to the clinician's instructions during the consultation. This means that several joints could have undergone medical imaging investigation, and this might result in medical imaging findings in other joints than the ones tested by the FT. This point was not addressed in this study only focusing on the presence of a diagnostic on a joint tested with the FT method.

A second limitation regarding the patient profile should be outlined: a large majority of patients in this population were under 1 year old. This could be linked with the proportion of patients more likely intact than neutered in this population. An explanation for this relative bias in the patient profile could be that elbow dysplasia cases are represented in a large majority as previously mentioned, and those cases are often diagnosed under 1 year of age (30).

Third, as mentioned before, this study has a retrospective design. This means the selection criteria are the same for all patients, but all examinations and lameness scores were not assessed on all patients by the same veterinarian to consider the FT positive or negative. Subjective lameness scoring is described with the use of a visual analog scale. This method implies inter- and intra-observer reliability (31). The absence of objective gait analysis methods (such as force plate or pressure sensitive walkway) before and after performing the FT can represent a lack of objective lameness measurements (32). Studies have shown that subjective lameness grading present low inter-observer agreement as well as agreement with objective gait analysis (33, 34). The studies about the FT available at this moment are not describing this specific point in enough details to state about a potential inter-observer reliability in the FT method (23). Therefore, lameness assessment requires careful observation and should always be interpreted with caution. Outcome assessment and patient positioning to perform the test are prone to variability depending on the examiner performing the technique (positioning and force applied) (23). Additionally, individual examiner's bias (such as the aggressivity of rating and the non-blinded status of the scorer to the painful joint) when rating outcomes were not quantified in this study and could be an additional confounder. Unfortunately, the data regarding the identity of the operator performing the FT was not available in this data set, and this prevents the authors to measure to potential influence of this parameter on the FT results.

Furthermore, dogs with severe lameness upon presentation (grade 4/4) were already maximally scored prior to the FT and could not score higher on the addressed outcome measures regardless of their lameness increase after the FT. This resulted in a misleading conclusion that the FT is less sensitive in patients with severe lameness. This further highlights the weakness of a four-point subjective lameness scale.

Regarding the FT method performed, a fourth described limitation is the lack of a standardized force evaluation. Equine lower limb studies suggested the force applied during the flexion may influence the outcome of the test (8, 9). A force of 100 N is mentioned in the equine species (9), however it is not measured routinely in equine practice and this might also be not technically possible on dogs. Further research is needed to determine exact force determination and support the repeatability of this test.

Finally, a remark should be brought up to the attention of the reader: this retrospective study focuses only on dogs known with an orthopedic disease where the authors decided to explore if the FT, when applied on this population of dogs, could be of any added clinical value. Therefore, this manuscript does not mention any false positive or true negative results. This creates a bias in unknown proportions. According to a previous study and clinical experience, these situations might occur (23), but further research is needed to understand the exact circumstances of these phenomena.

## Conclusion

Results were consistent with previous results available about the FT when applied on dogs. The canine FT is a technique easy to perform that may add value to the current orthopedic examination.

None of the dog physiological-related factors influenced the outcome of the test (age, sex, neutered status, and weight category).

In contrast, the orthopedic status of the dog, including the lameness score at presentation and the tested joint, influences the outcome of the FT. A dog with a mild or moderate lameness tends to present a greater measurable response to the FT than dogs without lameness or in severely lame cases. The type of tested joint also demonstrated an important influence, including higher reliability for digits, carpus, elbow, shoulder, tarsus and stifle compared to the hip joint which seems to produce slightly less reliable results when using this test.

As a conclusion, the flexion test can be applied to any dog regardless of his patient profile. A positive FT result should be considered as an indication for medical imaging investigation of the tested area.

## Data availability statement

The raw data supporting the conclusions of this article will be made available by the authors, without undue reservation.

## Author contributions

DG: study design, data collection, writing, and figures editing. ED: study design and writing. FF: study design, data exploitation, and reviewing the manuscript. AM: statistical analysis. JS: data collection and exploitation. BV and YS: study design and editing the manuscript. All authors contributed to the article and approved the submitted version.
